# Supplementary material for: Openness and COVID-19 induced xenophobia: The roles of trade and migration in sustainable development
Source: PLoS One. 2021 Apr 8;16(4):e0249579. doi: 10.1371/journal.pone.0249579 (PMC8031448; doi:10.1371/journal.pone.0249579)
Supplement: S1 Questionnaire — (PDF) [file pone.0249579.s001.pdf]

应对新型冠状病毒肺炎的海外华人华侨调查

尊敬的海外同胞和华人华侨朋友们：您们好！

针对当前新型冠状病毒肺炎现在国内爆发，随后遍及几乎全世界，这次疫情不但牵动着全球华人的心，同时也或多或少的对海外华人产生了直接或间接的影响。为更好的应对疫情，了解您的关切，以及支持国家的决策，由教育部人文社科重点研究基地云南大学西南边疆少数民族研究中心牵头开展这次调查，您的认真回复将有助于我们提出建议。我们的问卷需要**6 分钟**左右，我们将按照相关法律规定，严格采取匿名原则，保证您个人信息的安全，请您放心。

云南大学西南边疆少数民族研究中心

2020 年 2 月 9 日

1. 您是否关注国内最近发生的新型冠状肺炎疫情
   A.非常关注　B.比较关注　C.不太关注　D.没听说过

2. 你关心国内新型冠状肺炎疫情的原因是（多选）：
   A.亲戚朋友在国内　B.今年有回国内的行程安排　C.和自己的工作相关
   D.担心自己和家人被传染　E.热爱自己的祖国，　F=关心时事
   H=其他________

3. 目前您最关心的问题是（最多选两选）：
   A.国内的疫情发展蔓延的情况，　B.国内疫情控制政策变化，　C.居住国的疫情发展，
   D.您目前居住国控制疫情的政策变化，　E.特效药开发情况，　F.个人防护措施，
   G.本地是否有针对华人的过激行为，

4. 您是何时首次认识到新型冠状病毒肺炎疫情的
   A.12 月底　B.1 月初　C.1 月中旬　D.1 月下旬　E.2 月初　F.至今还不知道

5. 您获取国内疫情的信息渠道主要是？（多选题）
   A.国内的中央媒体　B.国内的地方媒体　C.商业网站　D.微博大 V
   E.微信公众号　F.公益组织　G.政府部门发布　H.医学专家
   I.专业疾病防控机构　J.熟人　K.BBC　L.CNN
   M.目前居住国的主流媒体　N.Facebook、tweet 等国外社交媒体　O.其他_________

6. 您认为国内疫情最可靠的信息来源是？请选择最可靠的三个来源________
   A.国内的中央媒体　B.国内的地方媒体　C.商业网站　D.微博大 V
   E.微信公众号　F.公益组织　G.政府部门发布　H.医学专家
   I.专业疾病防控机构　J.熟人　K.BBC　L.CNN
   M.目前居住国的主流媒体　N.Facebook、tweet 等国外社交媒体　O.其他_________

7. 您认为下列有关疫情防控的说法是否正确？

1.只有 N95 口罩才能有效防护病毒传播	A 正确　B 错误
2.吃维生素 C 能预防新型冠状病毒感染	A 正确　B 错误
3.燃放烟花能遏制呼吸道疾病	A 正确　B 错误

4.喝高度白酒能够有效遏制病毒	A 正确　B 错误
5.新型冠状病毒对热敏感，56°C 以上热水煮 30 分钟能够有效灭活病毒	A 正确　B 错误
6.75%以上的酒精、含氯消毒剂、过氧化氢消毒液等均可有效灭活病毒	A 正确　B 错误
7.此次病毒的致死率比 SARS 低	A 正确　B 错误

8. 您认为中国政府的疫情防控措施是否及时
   A.非常及时　B.比较及时　C.比较滞后　D.非常滞后

9. 您认为中国政府对疫情的防控措施是否有效？
   A.非常有效　B.比较有效　C.效果不太好　D.效果很不好

10. 您目前所在的国家或地区：________________

11. 您在目前的居住国的身份是：
    A.取得该国国籍（该国公民）　B.拿到该国绿卡(永居身份)
    C.长期移民（工作许可）　E.中国机构派驻海外人员
    D.留学生　，F.在中国国内有工作，临时在海外
    J.其他__________

12. 您已经在当前居住国居住的时间：
    A.6 月以下　B.6 个月-1 年　C.1-3 年　D.3-5 年　E.5 年以上，

13. 您出国定居年份大约在：
    A.1990 年以前，　B.1991-2000 年　C.2001-2010 年　D.2010 年以后
    F.还没有在国外定居

14. 疫情发生后，您的居住国或地区是否采取了限制中国人入境的措施：
    A.有　B.没有　C.不知道，

15. 对所在居住国限制中国人入境，您的看法是：
    A.应该限制，B.完全没必要限制，C.如果做好检查和跟踪，不应限制，　D.不好说

16. 在国内疫情发生后，您生活工作的环境有无歧视华人的情况
    A.有　B.没有(跳至 18 题)　C.不清楚

17. 国内疫情发生后，您生活工作的环境歧视华人的情况是：
    A.媒体有污辱中国人的信息　B.企业突然无理由解雇华人　C.房东拒绝租房子给华人
    D.公共场合不准华人进入　E.碰面避开　D.其他__________

18. 疫情发生后，您是否担心您的孩子在居住国受到歧视：
    A.非常担心，　B.有点担心，　C.一点都不担心，　D.我还没有孩子

19. 最近您周边是否发生了由于疫情而针对华人的过激行为：
    A.非常多　B.偶尔　C.没有

20. 由于疫情还在蔓延，如果您遇到针对华人的不愉快的事情或过激行为时，您会选择如何做(多选)：
A.以牙还牙， B.尽量避开，减少麻烦， C.报告和求助当地政府，
D.报告和求助中国大使馆 E.在社交媒体上曝光， F.提醒其他华人群体中注意，
G.不好说

21. 您所在国家是否有海外华人捐资捐物支援国内抗“疫”？
A.有， B.没有 C.不清楚

22. 您是否参与以下对国内疫情防控的捐赠
A.参与组织了对国内疫区的捐赠 B.自己给疫区捐赠 C.寄给亲戚朋友防护用品
D.没有以上捐赠行为

23. 您的籍贯或家乡在中国哪个省份？____________省/自治区/直辖市

24. 在疫情发生后，您是否与湖北籍人士有过交往：
A.有， B.没有， C.我身边没有湖北籍人

25. 您的性别：A.男 B.女

26. 您的年龄段是：
A.15 岁以下 B.16-20 岁 C.21-30 岁 D.31-40 岁
E.41-50 岁 F.51-60 岁 G.60 岁以上

27. 您的教育程度是？
A.小学 B.初中 C.高中、中专或技校 D.大学 E.研究生及以上

28. 您的最高学历是在哪里完成的? A.中国大陆 B.港澳台 C.国外

29. 您平时主要的出行方式是：
A.自驾车 B.步行 C.公共交通

30. 目前您出门是否会带口罩： A.会（跳至 32 题） B.不会

31. 您不戴口罩的原因是？
A.没那么紧张，不需要 B.居住国没有口罩文化 C.怕受到歧视或区别对待

32. 您最近是否会因为疫情而有意识的减少您的外出和交友活动：
A.会（跳至 34 题） B.不会

33. 您减少外出或交友活动的原因主要是：
A.避免遇到歧视或者其他麻烦 B.怕传染到病毒， C.其他_________

34. 在疫情发生后，您是否感觉周围的**本地人**和您接触时较之前更谨慎？
A=非常多　B=偶尔　C=没有

35. 在疫情发生后，您是否感觉周围的**中国人**和您接触时较之前更谨慎？
A=非常多，B=偶尔，C=没有

36. 您的职业是
A.专业技术人员（包括医生、教师、律师、会计师等） B.自营企业主　C.商人
C.企业管理人员　D.企业普通员工　E.农林牧副渔从业人员　F..国家雇员
G.自由职业者　H.学生　I.无业

37. 您在居住国工作的单位、学校或其他相关部门是否有下列措施或要求？（多选题）
A.要求所有人报告最近去往中国的旅行史，
B.要求华人报告最近的中国旅行史
C.要求近期有中国旅行史的所有人员开展自我隔离
D.要求近期有中国旅行史的华人开展自我隔离
E.减少或停止单位近期的对华往来
F.无以上措施或要求

38. 您所在的单位、学校或其他部门是否为减少“歧视”采取过任何措施（如发表非歧视声明等）：　A.有过　B.没有过

39. 您认为疫情爆发与中国人的生活习惯有没有关系：
A.有　B.没有（跳至 41 题）　C.不知道（跳至 41 题）

40. 您认为中国人如下生活习惯导致疫情爆发（可多选）：
A.爱吃野生动物　B.人际交往频繁　C.春节家人团聚　D.喜欢扎堆
E.合餐制　F.不注重个人卫生　G.其他

41. .您认为国内疫情扩散的主要原因是（可多选）：
A.没有及时公开疫情信息　B.公共卫生资源不足　C.公众防疫意识淡漠
D.医疗技术落后　E.防控措施不及时　D.其他___________

42. 您认为您现在居住国哪些疫情防控措施值得中国借鉴：
A.及时披露真实信息揭露　B.检查与治疗技术先进　C.防控措施有力
D.应急响应机制高效　E.公众卫生知识普及　F.其他__________

43. 您认为疫情对中国经济社会的影响短期内能够恢复吗？
A.能　B.很难　C.不能　D.不知道

44. 您感觉疫情对您的居住国经济社会的影响
A.影响很大　B.有影响但不大　C.没有影响　D.不知道

45. 您给提高国内疫情防控效率和完善公共卫生体系的建议是（开放性问题）：

Responds to COVlD-19 Overseas Chinese Survey[1]

Dear overseas compatriots and overseas Chinese friends: Hello!

In view of the current outbreak of Covid-19 in China and then all over the world, this epidemic not only affects the hearts of the global Chinese, but also more or less has a direct or indirect impact on overseas Chinese. In order to better respond to the epidemic, understand your concerns, and support the country's decision making, the National Centre for Borderland Ethnic Studies in Southwest China, Yunnan University,, a key research base of humanities and social sciences of the Ministry of Education, is leading this survey. Your serious reply will help us to put forward suggestions. Our questionnaire will take about 6 minutes. We will strictly adopt the principle of anonymity in accordance with relevant laws and regulations to ensure the safety of your personal information. Please rest assured.

National Centre for Borderland Ethnic Studies in Southwest China, Yunnan University,

February 9,2020

1. Are you concerned about the recent outbreak of COVlD-19 in China

A.Very much B.More C.Less D.Never heard of

2. The reasons you are concerned about the domestic Covid-19 epidemic are (multiple choice):

A. Relatives and friends in China

B. Travel arrangements back to China this year

C. It's about your job

D.Worry about the infection of oneself and family

E. Love one's motherland

F. Care about current events

H.Other

3.The questions you are most concerned about at this point are (choose two at most):

A. Development and spread of the domestic epidemic,

B. Changes in domestic epidemic control policies,

C. The development of the epidemic in the country of residence,

D. Changes in epidemic control policies in your current country of residence,

E. Development of specific drugs,

F. Personal protection measures,

G. Whether there are any aggressive acts against Chinese in the local area,

4. When did you first become aware of the Covid-19 outbreak

A.Late December

B.Early January

C.Mid-January

D. Late January

E. Early February

F. I don't know yet

---

[1] This questionnaire is directed translated from Chinese Version, which was used in survey. Please refer to Chinese version questionnaire for any misunderstanding from English Version.

5. What is the main channel for you to obtain information about the domestic epidemic situation? (Multiple choice)
A. The national media
B. The local media
C. The commercial websites
D. Microblog big V
E. We Chat Official Account
F. Public Welfare Organization
G. The government department issued an
H. Medical expert
I. Professional disease prevention and control institutions
J. Acquaintances
K.BBC
L.CNN
M. Mainstream media
N. Facebook, Tweet and other foreign social media in the current country of residence
O. Other

6. What do you think is the most reliable source of information about outbreaks in the country? Please select the three most reliable sources
A. The national media
B. The local media
C. The commercial websites
D. Microblog big V
E. We Chat Official Account
F. Public Welfare Organization
G. The government department issued an
H. Medical expert
I. Professional disease prevention and control institutions
J. Acquaintances
K.BBC
L.CNN
M. Mainstream media
N. Facebook, Tweet and other foreign social media in the current country of residence
O. Other

7.Do you think the following statements about epidemic prevention and control are correct?

1. Only N95 masks are effective in preventing the spread of the virus	A. True B.False
2. Taking vitamin C can prevent novel coronavirus infection	A. True B.False
3. Setting off fireworks can curb respiratory diseases	A. True B.False
4. Drinking high levels of liquor can effectively curb the virus	A. True B.False
5.Novel coronavirus is sensitive to heat. Boiling in hot water above 56'C for 30 minutes can effectively inactivate the virus	A. True B.False

6.More than 75% alcohol, chlorine-containing disinfectant, hydrogen peroxide disinfectant and so on can effectively inactivate the virus	A. True B.False
7. The fatality rate of this virus is lower than that of SARS	A. True B.False

8. Do you think the Chinese government has taken timely measures to prevent and control the epidemic

A. Very timely B. Relatively timely C. Relatively late D. Very lag

9. Do you think the prevention and control measures taken by the Chinese government are effective?

A. Very effective B. Relatively effective C. Not so good D. It doesn't work very well

10. Your current country or region:

11. Your status in your current country of residence is:

A. Obtain the nationality of that country (citizen of that country)

B. Obtain the green card of that country (permanent resident status)

C. Long-term Migration (Work Permit)

D. International students

E. People stationed overseas by Chinese institutions

F. Have a job in China, temporarily overseas

J. Other

12. How long have you lived in your current country of residence:

A. Less than 6 months B. 6 months -1 year C. 1-3 years D. 3-5 years E. More than 5 years

13. When did you go abroad to settle down?

A. Before 1990 B. 1991-2000 C. 2001-2010 D. After 2010 E. Not yet settled abroad

14. Since the outbreak of the epidemic, has your country or region taken any measures to restrict the entry of Chinese people?

A. Yes B. No C. Don't know

15. What is your opinion on the restriction of entry of Chinese people in the country of residence?

A. It should be restricted B. It is not necessary at all C. It should not be restricted if it is properly checked and tracked D. Hard to say

16. After the outbreak of the epidemic in China, have you been living and working in an environment that discriminates against Chinese people

A. Yes B. No (skip to 18) C.Don't know

17. After the outbreak of the domestic epidemic, you live and work in the environment of discrimination against Chinese is:

A. The media has insulting information about Chinese people

B. The company suddenly dismisses Chinese people without any reason.

C. The landlord refused to rent the house to the Chinese
D. Chinese are not allowed to enter public places.
E. Meet and avoid
F. Other

18. Are you concerned that your child will be discriminated against in the country of residence after the outbreak:
A. Very Worried B. Slightly Worried C. Not Worried at All D. I don't have any children yet

19. Has there been any violent behavior against Chinese in your neighborhood recently due to the epidemic?
A. Very much B.Occasionally C. No

20. As the epidemic is still spreading, what will you do if you encounter something unpleasant or aggressive against Chinese (multiple choice):
A. An eye for an eye
B. Avoid as much as possible to reduce the trouble
C. Reporting and seeking help from local authorities
D. Report and recourse to the Chinese Embassy
E. Exposure on social media
F. To remind other Chinese communities that
G. Hard to say

21. Have any overseas Chinese in your country donated money and materials to help fight the epidemic?
A.Yes B.No C. Not sure

22. Do you participate in the following donations for domestic epidemic prevention and control
A. Participated in organizing the donation to the domestic epidemic area
B. Donated to the epidemic area by oneself
C. Send protective equipment to friends and relatives
D. None of the above donations

23. Which province is your native place or hometown in China?

province/autonomous region/municipality directly under the Central Government

24. Have you had any contact with people from Hubei since the outbreak of the epidemic:
A. Yes B.No C. No, I don't know anyone from Hubei

25. Your gender: A. Male B. Female

26. Your age is:
A. Under 15 years old

B. 16-20 years old
C. 21-30 years old
D. 31-40 years old
E. 41 to 50 years old
F. 51 to 60 years old
G. 60 years old and above

28. Where did you complete your highest degree?
A. Mainland China B. Hong Kong,Macao and Taiwan C. Overseas

29. Your main ways of travel are:
A. By car B. By foot C. By public transportation

30. Do you wear a mask when you go out at present: A. Yes (skip to 32) B. No

31. What is your reason for not wearing a mask?
A. Not so nervous, don't need
B. The country where you live doesn't have mask culture
C. Fear of discrimination or differential treatment

32. Will you consciously reduce your going out and making friends because of the epidemic recently?
A. Yes (skip to 34) B. No

33. The main reasons for you to reduce your activities of going out or making friends are:
A. To avoid discrimination or other troubles B. To avoid catching the virus C. Other

34. After the outbreak, did you feel that the locals around you were more cautious in approaching you than before?
A. Very many B. Occasionally C. None

35. After the outbreak, did you feel that the Chinese people around you were more cautious in approaching you than before?
A. Very much B. Occasionally C. None

36. Your occupation is
A. Professional and technical personnel (including doctors, teachers, lawyers, accountants etc.)
B. Self-employed business owners
C. The merchant
D.Enterprise managers
E. Enterprise ordinary employees
F. Agriculture, forestry, animal husbandry and sideline fishery practitioners
G. State employee
H. Freelancer
I. Student

J. Unemployed

37. Does your work unit, school or other relevant department in your country of residence have the following measures or requirements?(Multiple choice)
A. Ask everyone to report their recent travel history to China,
B. Chinese are required to report their recent travel history to China
C. Require all persons with recent travel history to China to conduct self-quarantine
D. Requiring Chinese with are cent history of travel to China to self-quarantine
E. Reduce or stop the Company's recent dealings with China
F. No such measures or requirements

38. Has your organization, school or other department taken any measures to reduce "discrimination" (such as issuing A non-discrimination statement):A. Yes B. No

39. Do you think the outbreak has anything to do with the living habits of Chinese people?
A. Yes B. No (skip to 41) C. Don't know (skip to 41)

40. In your opinion, the following lifestyle habits of Chinese people have contributed to the outbreak (choose more than one):
A. Eat wild animals
B. Interact with people
C. Get together
D. Have A family reunion during the Spring Festival Like to gather together
E. Joint meals
F. Not paying attention to personal hygiene
G. Others

41. .In your opinion, the main reasons for the spread of the epidemic in China are (multiple choices):
A. Failure to disclose epidemic information in time
B. the lack of public health resources.
C. Public awareness of epidemic prevention is weak
D. Medical technology is lagging
E. Control measures are not timely
F. Other

42. What epidemic prevention and control measures do you think China should learn from your current resident country?
A. Timely disclosure of true information
B. Advanced examination and treatment techniques
C. Strong prevention and control measures
D. Efficient emergency response mechanism.
E. Popularization of public health knowledge. Other
F. Other

43. Do you think the impact of the epidemic on China's economy and society will be recovered in the short term?

A. Can B. Difficult C. Can't D. Don't know

44. You feel the economic and social impact of the epidemic in the country of residence

A. It has a great impact

B. It has an impact but not much

C. It has no impact

D. I don't know

45. Your suggestions for improving the efficiency of domestic epidemic prevention and control and improving the public health system are (open-ended question):
