# Supplementary material for: Openness and COVID-19 induced xenophobia: The roles of trade and migration in sustainable development
Source: PLoS One. 2021 Apr 8;16(4):e0249579. doi: 10.1371/journal.pone.0249579 (PMC8031448; doi:10.1371/journal.pone.0249579)
Supplement: Attachment — Submitted filename: Plos one_review December 2020.docx [file pone.0249579.s003.docx]

**Openness and Covid-19 induced xenophobia: the roles of trade and immigration in sustainable development**

This paper investigates whether more open economy in terms of trade and migration has an impact on observed discriminatory behaviors empirically. The theoretical frameworks build on existing research demonstrating that openness to trade and immigration facilitates more interactions with people outside one’s race, ethnicity and nationality, thus potentially improves mutual understanding and cultural exchanges and mitigating xenophobic sentiments. Using survey data on ethnically Chinese residents’ xenophobic experiences living abroad at the early stage of the pandemic in February, 2020, the authors find that greater openness to trade decreases the likelihood of reported xenophobic behaviors while openness to migration increases it.

I think that the paper will be interesting to scholars in political economy and public opinion. The paper addresses an interesting but unexplored question. I recommend revisions before the paper appears in *Polis-one* or another journal.

1. Theory

The objective of this paper is to examine whether there is a causal effect of trade and immigration policy on discriminatory behaviors observed by ethnically Chinese residents living abroad at the early time of the covid-19 pandemic. Although the paper is empirical-oriented one, we still need some theoretical explanations of how changes in trade and immigration affect discriminatory behaviors observed. In this sense, the paper has some issues to be addressed. First, it does not explain why we focus on the effects of immigration and trade on discriminatory behaviors observed. Globalization can have various dimensions such as trade, foreign direct investment, immigration, and capital market liberalization. Given the various aspects of globalization, I am wondering why the authors pay particular attention to the two dimensions of globalization – trade and immigration, not trade and FDI or trade capital market liberalization, etc. The authors would justify this point well in the main text.

Second, I am wondering how trade and immigration influence discriminatory behaviors observed among ethnically Chinese residents living abroad. I think that a large body of literature in international political economy has explained underlying mechanisms linking trade and immigration to xenophobic behaviors. Yet the authors do not present these mechanisms in a systematic way, while just describing some selective studies supporting their theoretical frameworks. In doing so, the authors do not describe whether trade and immigration have a negative, positive, or null impact on xenophobic behavior observed. The authors would elaborate some testable hypotheses on the effect of trade and immigration on xenophobic behavior observed.

2. Empirics

The authors predict that an increase in trade and immigration can have a negative, positive, or null effect on xenophobic behaviors observed. The questionnaire is “Since the virus outbreak in China, have you noticed any related discriminatory behavior in your working environment and daily life?.” The underlying logic behind the hypothesis is that openness to trade and immigration facilitates more interactions with people outside one’s race, ethnicity and nationality, thus potentially improves mutual understanding and cultural exchanges and mitigating xenophobic sentiments. Given this logic, how can we know that trade and immigration affect local natives in a way that the authors expect? To figure out the mechanisms, wouldn’t it be better to do some survey experiments to local natives rather than ethnically Chinese residents? Simply asking ethnically Chinese residents living abroad does not show that they identify the causal mechanisms between trade and immigration and xenophobic behaviors as local natives are main actors under their theoretical framework.

-Related to the above point, another concern is that the authors use trade and immigration indicators at the national level. Employing the national-level indicators capturing trade and immigration does not tell that individuals are affected by them. Although countries may be more economically opened in terms of trade and immigration, it does not necessarily mean that individuals know, experience, and perceive them objectively and subjectively. If the authors attempt to uncover the causal mechanisms, it would be better to use some survey questionnaires to measure how individual respondents know, experience, and perceive the degree of trade and immigration at the individual level.
